# Azoturia*

**Published:** 1885-04

**Authors:** Thomas W. Rogers


### Art. XII.—AZOTURIA.*

BY THOMAS W. ROGERS, V.S.

I purpose to-day to call your attention to Azoturia, the hæmoglobinuria of the Germans. The first notice of this condition in English speaking literature is found in Haycock's contributions to veterinary pathology, article Hysteria.

Haycock observed the disease only in mares, and was led to the conclusion that it was an abnormality of the sexual function somewhat analogous to nymphomania. Subsequent writers have shown that it is almost as frequent in horses as mares.

*Symptoms.*—These follow usually in a well-worn groove. The horse, after a number of days confinement, during which time he has received his usual allowance of highly nutritious food, is taken out to drive; he goes right up to the bit—indeed the driver often remarks that he could hardly hold him—then often without premonition he falls in the shafts, unable to rise with or without assistance. The animal, retaining all his powers of mind, exhausts himself in fruitless struggles to rise, breaks out into a profuse perspiration, and if urine is discharged during this time it is usually dark in color—coagulating rapidly on cooling. Sometimes this condition persists until death; at others the symptoms ameliorate almost as rapidly as they showed themselves. There is swelling of the glutei, hard and board-like in character. The horse is usually well on the road to recovery or dead in forty-eight hours. The temperature is elevated.

*Differential Diagnosis.*—The conditions liable to be confounded with azoturia are—apoplexy, simple paralysis due to nervous lesion, spinal and cerebro-spinal meningitis. From apoplexy it may be distinguished by the retention of the sensory functions, the absence of oral breathing and the character of the urine. From simple paralysis, by the fact that in azoturia neither sensation nor motion are totally in abeyance, and again by the characteristic urine. In paralysis caused by obstruction of the iliacs, examination of the aorta post and its quadrifurcation will establish the diagnosis, and abnormal coldness

* Read before the New Jersey Veterinary Medical Association, Dec. 10, 1884.

of one or both hind limbs will lead the practitioner to make such examination.

From cerebro-spinal meningitis it may be differentiated by the difference in temperature—in cerebro-spinal meningitis below normal or normal, in azoturia always elevated; the swelling of the quarter is absent in cerebro-spinal meningitis; and there is usually difficulty in deglutition, and often alterations in the higher sensory functions. The urine may be dark, but is usually of a redder tinge than in azoturia; microscopically the diagnosis is patent. We find bloody urine in cerebro-spinal meningitis, urine dark from the presence of free hæmoglobin or its derivatives in azoturia. In spinal meningitis—a rare condition—I have only seen four cases in a practice of five years—the symptoms are not usually fulminant. If the animal is in harness there are short periods of great lameness, amounting to mobility of progression, followed by the animal falling; there is a straddling, squatting gait, the symptoms much aggravated by rectal examination (this also in azoturia); the horse often gets up and down, has clonic spasms of the muscles of the entire body; these are very characteristic, as are also the intervals of ease; there is straddling and paddling of the hind legs as in kidney troubles, no hardness of the quarters, and usually little discoloration of the urine. The struggles when down are not at all confined to efforts to rise, as in the early stage of azoturia, but are made without reason, though not without rhyme, as they are usually rhythmical in character. Later on in azoturia we have, however, similar convulsive efforts.

From blind staggers it may be diagnosed by the difference in situation; blind staggers, probably anthracoid in character, is only seen in animals pastured on low, rich-bottomed meadows in summer time, azoturia is usually a disease of winter and of the stabled animal.

*Causation.*—Azoturia is a disease of the functions of nutrition—a disease of nutritive excess. It is a disease of the well-to-do man's horse.

One of the conditions of health is that the excess of nutrition be removed by the lymphatics; it is conveyed again to the right side of the heart along the thoracic duct, and again poured into the current of the circulation to nourish the tissues. In azoturia there is so great nutritive excess that a large portion of nutritive matter is thrown back, undergoes retrogade decomposition into urea, or the analogues of urea, and acts as a poisonous substance on the nerve centres. Centrally it is probably a failure of the liver to perform its scavenger-like function of burning up the waste proteids.

What I wish especially to call your attention to, however, is that whatever its exciting cause, *azoturia is uræmia.* You have to deal with uræmic poisoning, and whether this is caused, as in this case, by the impaction of a surplus of nutrition in a healthy, hard-working organism, or is due to inability of the kidneys to separate retrograde material from the blood, matters little as regards your treatment. The question may be asked, why is azoturia more common in mares than in horses? I think the question admits of ready answer. The female organism is, so to speak, more conservative than the male, intended by nature to care for and support her parasitic offspring. She lays up with greater readiness than does the male a supply of nutritive matter in excess of her own requirements. I think this part of my explanation is strengthened by the fact that mares in foal rarely have azoturia. Secondly, the function of reflex irritation is higher in mares than in horses. Dr. Zuill, of Philadelphia, informs me that he has found as high as 22 per cent. of urea in the urine of azoturia.

*Post Mortem appearances.*—Congestions of the meninges. The blood often chocolate-colored or somewhat tarry, but not abnormally fluid as in the tarry blood of anthrax, or deficient in red disks, as in the leucocythemia. The color is probably due to the admixture of the effete lymph. The liver is usually yellowish, enlarged, friable; the kidneys enlarged and congested, sometimes containing pus in their pelves, and the true mucous membrane of the bladder inflamed. The condition of the bladder is due to ammoniacal decomposition of the urine in a similar way to that found in old men with enlarged prostate, the constant action and reaction of the mucous thrown out by the irritated viscus on the urine, and the irritation of the urine assuring the secretion of the mucous.

I call your attention to a sequel of azoturia unmentioned, as far as I know, in English works. It is an almost complete atrophy of the crural triceps of the muscle of the fascia lata, and in some cases of the glutei of one side. The first case I saw was in Philadelphia. I was called to "*put in the stifle*" of a horse. I found the patella in its place, but imagined that I had a dislocation of the femero-tibial articulation. I was satisfied that I could place my hand on the tibial spine, and to show you that this great error of diagnosis was not due to any carelessness, two thoroughly qualified Philadelphia veterinarians agreed with me, and also agreed that attempts at reduction would be futile, as if, by extension and counter-extension on the limb while the horse was recumbent, the dislocation could be reduced, the condition would recur on rising. A big blister was placed on the affected side and the horse left to his fate. Imagine my surprise when I saw the horse last summer as sound as a dollar, without a trace of the old trouble.

Since then I have been consulted on several similar cases ; have advised as treatment repeated blisters and the battery, with a long run at grass. This has been successful in each case, but has required many months to bring about the result. The appearance is peculiar ; there is apparently a big hole in the affected flank, and the femur is almost denuded of musculature arteriorly.

Dr. Huidekoper suggested to me that rupture of the psoæ was partly the cause. This may be so in some cases, though none of mine had sufficiently marked abduction of the limb to warrant the diagnosis. The cause may be a tissue change of pyrexia, local embolism interfering with nutrition, or altered nervous action, due to pressure. One of my cases was complicated with a most distressing vesica vaginal catarrh.

*Treatment.*—Starting from the standpoint that we are dealing with uræmia, the endeavor of the intelligent practitioner will be addressed to ridding the system as quickly as possible of the poisonous matter. I well remember, when a student, seeing these cases brought in usually to be carried out again heels up. The routine treatment is to purge and bleed. The first part of the treatment I will pass over with the remark that if the patient would be accommodating enough to live until the purgative acted, it would be very good treatment indeed. Usually he dies before the purge acts. If he lives eighteen to twenty-four hours, and goes on to recovery, there is the open question as to whether the purge or the *vis medicatrix naturæ* is to be thanked for it. Bleeding is, I think, to be recommended; it offers a means by which we are enabled to rapidly remove a large amount of urea from the body, and also tends to lessen the action of the remaining poison on the nerve centres. To bleed and purge, however, is to knock down with one hand and set up with the other, as nature, ever tenacious of her balance, will rapidly withdraw from the body fluids enough to make up the mass of blood, and thus hinder the action of a purge, depending, as it does, for its action, in a great degree on the maxim, "ubi irritatio ibi affluxus." When I tell you that I rely almost exclusively on morphia, and that my practice has been so successful that last winter about nine cases, most of them bad ones, made good recoveries, it may provoke a smile from those of you who are intensely practical.

Let us see. What does morphia do for us? In the first place it puts a stop to the action of the urea on the nerve centres, it quiets the irritable patient, it allows him to stand quietly in slings and so avoid knocking the bark off himself in futile struggles. You may be still more incredulous when I tell you that my principal reason for the use of this remedy is to get its eliminative action—and you wont get this from the use of ten or twenty grains. You are of course aware that the narcotics may be pushed much further in the lower animals than in man; their action is directed more to the cord and less to the higher faculties than in man, and to get good results from the morphia treatment of azoturia you must push it far enough to get its toxic action. You must give ten or fifteen grains every hour or so until the patient rests quietly and sweats profusely. You may push it so far that it induces automatic, convulsive movements of the limbs, simulating trotting or galloping, and you will do no harm by this course. To reassure you, I may state that the same treatment is frequently adopted in the uræmia of mankind with equally good results. I have given a drachm in twelve hours without producing sleep and with excellent results. Do not be afraid of it, use it freely and it will do you good service.

With regard to other treatment, I advise you to get the horse into slings as early as possible; if he entirely loses the control of his hind limbs it will, of course, be necessary to lower him, and I may add that a smart stroke of the whip will often make him stand when hanging in them.

The local complication, the "kidney trouble," attracts usually most attention from the laity and the ignorant horse doctor.

Gentlemen, let me most strongly impress upon you the fact that it is not a kidney affection. The kidneys are doing their work—are working up to the very limit of their power. Do not aggravate them by the use of diuretics, especially of those resinous in character. If you must use them, then, and when on the road to recovery, use squills and digitalis. If there is retention, shown by examination of the bladder, you may pass the catheter. And here again a word. Do not do it until your horse is comfortably in slings and has had a dose or two of morphia, as I have on several occasions seen the irritation caused by the passage of the catheter cause convulsions and the getting down of the animal. Of course if you find your patient recumbent with a full bladder it is well, especially in the horse, to put on the hind hobbles and take away the water, though personally I prefer to pass the catheter standing.

A hint about catheter. You will find the thick foreign instrument, with bluntish point, much less liable to lose its way than the thin American one, fit only to pass on the stretched urethra of a bull.

Have you vaginitis or cystitis following? Treat them on general principles: rest the bladder by the use of purgatives, sheath it by the administration of mucilaginous drinks, soothe it by warm rectal injections, and you may, do you deem it necessary, supplement these means by washing out the bladder through the catheter. What food? No food for forty-eight hours; then food poor in nitrogen. Preventive measures, thorough ventilation, good drainage, good grooming, regular exercise,—even if the hired man has to be sent out sleighing,—and a diminished ration if the quietude is imperative. You will find the question as to the use of nerve stimulants and blisters discussed in the scanty and disgraceful veterinary literature of this subject.

Their use could only arise from a mistaken notion regarding the pathology of the condition, and I would take this opportunity of saying that if you would aid in the advance (now so great) of veterinary science in this country, you must go to comparative medical literature for aid. You are not competent to treat diseases of the lower animals unless you are able to thoroughly understand and appreciate human pathology. Confine yourselves to the scanty pabulum offered you by veterinary literature, and you will be routine practitioners at best, groping in the dark, not after science but dollars, and although the ability you may discover in finding these last may be a surprise to your neighbors and yourselves, you will never benefit your chosen profession one ha'porth, nay, you will aid to drag it down, to clip its wings and keep it where it has been too long, in the dog-eared volume of every man his own horse doctor, in the chest where the blacksmith keeps his spare nails and oakum, or the livery stable man his winter blankets.

If you can do no more, make a careful record of facts, and publish them every once in a while for those more favored by circumstances to draw inferences from, report your unsuccessful cases as well as those redounding to your credit, and so aid in suppressing the worm in the tongue, the wolf in the tail, and the salt mackerel for the loss of cud of our generation.

