# Correction

**Published:** 1885-07

**Authors:** 


Correction.—We desire to correct a typographical error making the author of the interesting article on "Azotura," published in our last Journal Thomas W. Rogers, instead of Thomas Balffe Rogers, of Westville, N. J. We are pleased to state that Dr. Rogers promises us an article on the diseases of the intestinal canal, to appear in the next number.

